# Incorporating African American Veterans’ Success Stories for Hypertension Management: Developing a Behavioral Support Texting Protocol

**DOI:** 10.2196/29423

**Published:** 2021-12-01

**Authors:** Kathryn L DeLaughter, Gemmae M Fix, Sarah E McDannold, Charlene Pope, Barbara G Bokhour, Stephanie L Shimada, Rodney Calloway, Howard S Gordon, Judith A Long, Danielle A Miano, Sarah L Cutrona

**Affiliations:** 1 Center for Healthcare Organization and Implementation Research VA Bedford Healthcare System Bedford, MA United States; 2 Boston University School of Medicine Boston, MA United States; 3 Nursing Ralph H Johnson VA Medical Center Charleston, SC United States; 4 College of Medicine Medical University of South Carolina Charleston, SC United States; 5 Population and Quantitative Health Sciences University of Massachusetts Chan Medical School Worcester, MA United States; 6 Department of Health Law, Policy and Management Boston University School of Public Health Boston, MA United States; 7 Jesse Brown Veterans Affairs Medical Center and VA Center of Innovation for Complex Chronic Healthcare Chicago, IL United States; 8 Section of Academic Internal Medicine Department of Medicine University of Illinois at Chicago Chicago, IL United States; 9 Institute for Health Research and Policy University of Illinois at Chicago Chicago, IL United States; 10 Corporal Michael J Crescenz VA Medical Center VA Center for Health Equity Research and Promotion (CHERP) Philadelphia, PA United States; 11 Division of General Internal Medicine Perelman School of Medicine University of Pennsylvania Philadelphia, PA United States

**Keywords:** texting, African American, hypertension, self-management, mobile phone

## Abstract

**Background:**

Peer narratives engage listeners through personally relevant content and have been shown to promote lifestyle change and effective self-management among patients with hypertension. Incorporating key quotations from these stories into follow-up text messages is a novel way to *continue the conversation*, providing reinforcement of health behaviors in the patients’ daily lives.

**Objective:**

In our previous work, we developed and tested videos in which African American Veterans shared stories of challenges and success strategies related to hypertension self-management. This study aims to describe our process for developing a text-messaging protocol intended for use after viewing videos that incorporate the voices of these Veterans.

**Methods:**

We used a multistep process, transforming video-recorded story excerpts from 5 Veterans into 160-character texts. We then integrated these into comprehensive 6-month texting protocols. We began with an iterative review of story transcripts to identify vernacular features and key self-management concepts emphasized by each storyteller. We worked with 2 Veteran consultants who guided our *narrative text message* development in substantive ways, as we sought to craft culturally sensitive content for texts. Informed by Veteran input on timing and integration, supplementary educational and 2-way interactive assessment text messages were also developed.

**Results:**

Within the Veterans Affairs texting system *Annie*, we programmed five 6-month text-messaging protocols that included cycles of 3 text message types: narrative messages, nonnarrative educational messages, and 2-way interactive messages assessing self-efficacy and behavior related to hypertension self-management. Each protocol corresponds to a single Veteran storyteller, allowing Veterans to choose the story that most resonates with their own life experiences.

**Conclusions:**

We crafted a culturally sensitive text-messaging protocol using narrative content referenced in Veteran stories to support effective hypertension self-management. Integrating narrative content into a mobile health texting intervention provides a low-cost way to support longitudinal behavior change. A randomized trial is underway to test its impact on the lifestyle changes and blood pressure of African American Veterans.

**Trial Registration:**

ClinicalTrials.gov NCT03970590; https://clinicaltrials.gov/ct2/show/NCT03970590

**International Registered Report Identifier (IRRID):**

DERR1-10.2196/29423

## Introduction

### Background

Uncontrolled hypertension is common among African Americans, leading to higher rates of cardiovascular morbidity and mortality [1]. High prevalence and inadequate control of hypertension in this group contribute to persistent disparities in stroke, heart failure, peripheral artery disease, and end-stage renal disease [2,3]. Disparities in hypertension control may derive from differential rates of diagnosis, treatment titration, and patient factors, including medication adherence, lifestyle changes, self-monitoring, and engagement with the health care system. Due in part to experiences of racial discrimination (personal and historical), African Americans routinely express lower levels of trust in the health care system. Thus, to promote sustained behavior change, hypertension self-management interventions designed to support African American patients must be personally relevant [4] and acknowledge and address mistrust in the health care system.

Peer narratives, also referred to as *storytelling*, can provide personally relevant messages, helping address mistrust and cognitive resistance arising from concerns about treatments or medical information [5]. In contrast to clinical recommendations that may be perceived as abstract or disconnected from daily experiences, peer narratives provide real-world examples of behavior embedded in daily lived experiences [6]. Peer interventions have been used to support African Americans in achieving effective hypertension self-management [7-10] and storytelling can play an important role in this support. When a patient is engaged in a story, their attitudes and intentions can be influenced by features from the story, and this engagement is often mediated by personal identification with the storyteller.

In our previous work, African American Veterans shared stories of struggles and strategies related to hypertension self-management, which we video-recorded [11]. We found that patients who viewed story videos were more likely than those who viewed an educational DVD to be engaged emotionally and report intent to change behaviors [12]. However, we found that this one-time intervention did not translate into a sustained improvement in blood pressure. Therefore, we sought to provide additional longitudinal support.

Text messaging has proven to be an effective and inexpensive way to communicate with patients longitudinally [13] and shows promising results as a means of supporting hypertension control [14]. A randomized feasibility texting intervention conducted among African Americans [15] with uncontrolled hypertension found trends toward improvements in medication adherence, as well as systolic and diastolic blood pressures. A larger text-messaging randomized trial addressing hypertension treatment adherence in over 1300 South Africans showed small but significant improvements in blood pressure after a 12-month intervention [14]. A small trial of text messaging paired with electronic medication tray reminders showed significant improvement in the number of African American and Hispanic participants who achieved blood pressure control after 6 months [16]. Several larger randomized trials are underway to further explore text messages as a means of supporting hypertension control for African Americans.

### Objective

In this paper, we describe the process by which we developed text message content and texting protocols aimed at incorporating the voice of African American Veteran storytellers [17]. Our goal is to extend the impact of our previous peer storytelling intervention by *continuing the conversation* through engaging, personally relevant text messages adapted from the Veterans’ video-recorded stories.

## Methods

### Overview

We designed our text messages for use in a Veterans Affairs (VA)–based text-messaging platform, *Annie* [18]. Our multidisciplinary team included physicians (SLC, HSG, JAL), sociolinguists (BGB, CP), anthropologists (GMF), experts in informatics (SLS, SLC), self-management behavior specialists (KLD, SEM), qualitative analysts (DAM, SEM, KLD), and African American Veteran consultants (Rodney Calloway and Paula Smith-Benson). We began text message development by identifying key concepts in hypertension self-management that emerged from our previously recorded Veteran stories. We then used these key concepts to guide the development of 3 distinct text message types (narrative, educational, and interactive). Throughout this process, we sought input from 2 African American Veteran consultants to improve the cultural sensitivity and relevance of narrative text messages to support our goal of designing messages that transmitted each Veteran voice with authenticity and respect. Social Cognitive Theory (SCT) [19] informed our approach to framing messages aimed at enhancing self-efficacy, with the goal of ultimately influencing health behaviors.

Below, we describe the texting platform we used and our approach to developing the 3 text message types: (1) *narrative* texts, (2) educational texts, and (3) interactive messages assessing self-efficacy and behavior related to hypertension self-management.

### Using the VA-Based Text-messaging Platform

*Annie* is an SMS text-messaging system in the Department of VA, which is available to support routine clinical practice. Providers can access a secure dashboard and choose from a menu of texting protocols intended to promote self-care for Veterans enrolled in VA health care. Veterans can receive text messages directly on their phones or through a VA mobile app downloaded into their smartphones. Existing texting protocols provide motivation, education, and, in some cases, an invitation to monitor the status of their chronic illness (eg, reporting blood sugar readings) [20]. The *Annie* text-messaging system is not intended to be monitored by providers and delivers an error message if free text is sent in by a Veteran (or if any response at all is sent in for noninteractive messages).

The *Annie* system contains templates for one-way and 2-way texts that allow for the customization of timing and content. Each message is limited to a maximum of 160 characters and must include the word *Annie* as an identifier. Short URLs can be included in the text. Response options for 2-way texts cannot be open-ended and must be preprogrammed into the system. We worked closely with a few VA researchers experienced in the use of this platform as well as with VA operational partners to understand the constraints imposed by *Annie* and to construct messages that optimized available opportunities.

### Key Content Area Identification

In our previous work, we created video-recordings of 5 stories (each about 5-8 minutes long), told by African American Veterans who had successfully managed their uncontrolled hypertension [12]. The development of these narrative videos has been well described previously [11] and included the identification of key content areas (Table 1). During that work, we sought to maintain authenticity and the voice of the storyteller, while also describing small specific behavioral action steps, bringing them together in a video-recorded story designed to engage and maintain viewer attention.

**Table 1:** Key content areas and description.

Key area	Description
Salt intake	Veterans describe strategies and contextually situated stories of how they managed salt intake
Talking with your physician	Veterans describe reasons why honest communication with providers is important
Take your medicine	Veterans describe strategies and motivation tools in taking their prescribed medicine
Exercise	Veterans talk about specific strategies to increase exercise
Stress management	Veterans describe tools and tactics to manage stress
Monitor your blood pressure	Veterans tell stories of ways they monitored their blood pressure
Diet or nutrition	Veteran storytellers describe how they improved their diet or learned more about the importance of nutrition
Faith or church or community	Veterans share how their faith, church or community is a source of support or motivation for them and their health
Alcohol or smoking or challenges	Veterans share their experiences with alcohol, smoking, etc and how they addressed these challenges

### Narrative Text Development

#### Overview

*Narrative text messages* are text messages that incorporate content from Veteran stories aligned with the key content areas outlined in Table 1. To develop content for the narrative text messages in this study, we sought to identify microstories within these longer narratives. We took a multistep approach beginning with (1) review of transcripts (GMF, KLD, SLC, SEM, CP, BGB, and SLS) from patient stories and selection of quotations aligned with previously identified key content areas, (2) followed by creation of 160-character messages capturing the Veterans’ voices (GMF, SEM, SLC, CP, KLD, BGB, and SLS), each aligning with a key content area [11]; and (3) finally, solicitation of feedback from Veteran consultants.

For example, (Table 2), we created a text message focused on the Key Content Area of Faith or Church or Community as follows: In step (1), a Veteran storyteller’s transcript captures his description of a wish to *give back* to other Veterans and contribute to his community because *I’ve been through it too*. Although this message is not specific to hypertension self-management behaviors, it was chosen for its potential to reinforce the recipient’s identification with the storyteller and to strengthen the message of this key content area, which focuses on gaining support for healthy behaviors through one’s social network. In step (2), we identified and extracted a few words that exemplified this message. In step (3), the Veteran consultant reviewed this message during a feedback session and suggested modifications.

**Table 2:** Examples of narrative text messages (adapted from quotations from African American Veterans sharing stories about how they manage their hypertension).

Hypertension self-management key content area	Direct quotation from unedited video transcript^a^	Draft text message	Examples of ways that narrative content and Veteran input were used to modify texts	Final
Low sodium diet	“*Basically, I don’t have any salt, plain salt in my house. S-salt substitute, seasoning*, uh, a low salt, *anything with, that’s low salt* in there**,** *I use that,* you know. It’s not as strong as the salt that I usta take but still crave, you’re given that, all, *keep me from craving for regular salt,* you know.”	“*Annie-BP*: Willie says-basically I don’t have any salt, plain salt in my house. Salt substitute, seasoning, anything that’s low salt-I use that.”	Informed by narrative content, we incorporated the concept of craving The Veteran used this concept several times in the narrative to emphasize the role played by salt substitute	“*Annie-BP*: Willie says-basically I don’t have any plain salt in my house. Salt substitute or seasoning keeps me from craving for regular salt.”
Faith or church or community	“*I’m a Veteran tryin’ to give back to a Veteran***.** If I’m, they-they gave to me, you know, a new, a-await in life, you know, but homeless vets, the Veterans are homeless out here today and my thing is to try to do for our homeless myself at one time and, uh, uh, different things I went through in life, *I can basically relate to the ‘nother Veteran because I’ve been through it, too***.** So that’s what I do now is house homeless vets and then we have pro-, uh, programs for, like posttraumatic stress and we all talk about PTSD.”	“*Annie-BP*: Willie says: I’m a Veteran trying to give back to a Veteran. I can relate because I’ve been through it too.”	Informed by Veteran consultant feedback, we deleted: I can relate because I’ve been through it too. Our consultant felt this would be understood by another Veteran without needing to be stated.	“*Annie-BP*: Willie’s doing something he loves to do while helping others in the community. Willie says: I’m a Veteran trying to give back to a Veteran.”
Exercise	“I began to see that it was affectin’ a lotta things in my life so I decided to do somethin’ about it, and that’s why I call it a journey...That’s what I do. *I do little stuff that adds up to big stuff...And I do it every day,* cuz like I said, there’s not, it’s nothin’ strenuous, you know what I mean?...I do a whole lotta little stuff that I think adds up to somethin’ big, you know...I do, *I do a lotta little stuff that adds up to big stuff, you know.*”	“*Annie-BP*: Richard talks about his BP: it was affectin’ a lotta things in my life so I decided to do somethin’ about it. I do a lotta little stuff that adds up to big stuff.”	Informed by Veteran consultant feedback, we adopted standard spelling, while trying to keep the cadence of the Veteran voice. Our consultant observed that professionally typed transcriptions can reflect the bias of the transcriber. Including nonstandard spelling may offend those who personally experienced stigmatization related to their speech patterns.	“*Annie-BP*: exercise doesn’t have to mean a big lifestyle change. Richard says, I do little stuff that adds up to big stuff and I do it every day.”

^a^Italicized text corresponds to sections either paraphrased or directly quoted in text message.

#### Review of Story Transcripts and Selection of Quotations

We began our text message development by iteratively reviewing the transcripts of video-recorded Veteran stories. Two study team members reviewed the 5 unedited video transcripts (one from each Veteran storyteller). The transcript review included (1) identification within each story of key concepts in hypertension self-management and key narrative elements; and (2) selection of longer transcribed segments containing quotations conveying these key hypertension concepts, key narrative elements, or linguistic phrases characteristic of the storyteller. These longer (usually multi-sentence) excerpts from storyteller transcripts were then categorized based on key concepts. We initially selected and categorized longer segments because we wanted to ensure that we captured the full context of quotations. We did this to avoid misrepresenting the intended idea when (in subsequent steps), we pared down the wording to fit in a brief text message. In some cases, longer excerpts were categorized as relevant to multiple key hypertension self-management concepts, and text messages were adapted from these for more than one key concept area.

#### Creation of 160-Character Text Messages

In these longer segments, we then highlighted smaller sections of quoted text that contained key story elements, key hypertension self-management concepts, or linguistic phrases that had either been repeated throughout the story or were evocative of the storyteller’s unique personal style. We sought concepts that could be succinctly communicated and examined. We considered whether we could delete text to shorten the message without distorting the intended meaning and whether there were ideas that would be better communicated by paraphrasing because of the structure of the quotation.

We began drafting potential text messages based on this review, often writing out 2 or more possible versions. As our ultimate goal was for these text messages to be delivered after study participants watched the 5-to 8-minute video-recorded versions of our storytellers, we also noted whether the excerpted text reflected a section that had been included in the final edited video (although we did not require that text messages be derived solely from segments included in the video).

Our multidisciplinary team met weekly as a group to review the text messages that were created, identified favorites by consensus, and edited iteratively for character count and flow. Our Veteran consultants also met with a team member separately to allow for more in-depth feedback. The diversity of training and expertise on this team enhanced our ability to look at the nuances of texts and their implications. Some video-recorded Veteran storytellers did not address all the key concepts (or the storyteller addressed the concept in language, not lending itself to incorporation into a 160-character narrative text). Our planned intervention requires participants to watch all 5 videos, and then select a single storyteller they prefer. This choice will then inform assignment to the corresponding texting protocol, which will include quotes from the participant’s preferred storyteller. Our goal was to cover all the key concepts for each storyteller protocol. Therefore, we used narrative messages from another Veteran storyteller to cover the missing concepts. When we performed this step, we included wording to distinguish the quotation. For instance, instead of *Morris said* we used *a Veteran who shared his story with us said:* as in the example: “ANNIE-BP: *A Veteran who shared his story with us said*: I was enjoying the smoke but also I was hurting myself at the same time. If I wanted to live, I had to give up cigarette smoking.”

#### Feedback From Veteran Consultants

We invited one of our consultants (coauthor RC) to view storyteller videos and give inputs for our process of creating texts. Our goal was to design messages that conveyed each Veteran’s voice with authenticity and respect. As an African American Veteran himself, RC guided us in our efforts, helping us improve the cultural sensitivity and relevance of our narrative text messages.

We asked RC for reactions to individual storytellers and enquired which story segments and themes were most memorable. These responses helped inform the selection of quotations from the unedited transcripts. RC reviewed drafts of narrative texts as we created them and guided our choices on (1) language structure, (2) word choice and tone, and (3) fidelity to storyteller voice. For each of the 5 storytellers whose stories were developed into text messages, RC met with us for multiple 1-hour periods over several weeks, reviewing every narrative text message. For an additional perspective, we invited our second Veteran consultant (Paula Smith-Benson) to provide additional feedback. Her input was focused on a story told by our female Veteran storyteller; therefore, Ms Smith-Benson reviewed only a subset of narrative messages.

### Educational and Interactive Text Development

Alongside our narrative text messages, we developed educational and interactive text messages corresponding to each key content area.

#### Educational Messages

Educational messages incorporated content from the American Heart Association, the American College of Cardiology, and the Centers for Disease Control and Prevention [21]. Each message focused on one of the key content areas outlined in Table 1 (eg, for salt intake: “ANNIE-BP: You can also reduce salt by avoiding the saltshaker. Avoid sea salt and garlic salt too—try substitutes instead: Mrs. Dash or fresh herbs for flavor”).

#### Interactive Text Messages

Interactive text messages were 2-way messages that asked a question, prompted a reply, and sent a follow-up comment. These had several purposes: motivation, patient engagement, data collection, and education. We adapted questions from validated scales [22,23] where available. Guided by SCT [24], we assessed (1) participants’ confidence in their own ability to carry out a hypertension self-management task (self-efficacy) and (2) participants’ actions toward effective self-management (behavior). Interactive text messages often provided content-specific resource links to educational sites (eg, the American Heart Association) or VA webpages.

Interactive text responses were developed using preset phrases (Table 3). For self-efficacy: “How confident are you that...Text NA 1 (not confident), NA 2 (somewhat), or NA 3 (very) to reply.” For behavior performed across a number of days per week: “How many days this week have you...Text ACT 0, ACT 1 up to ACT 7 to reply.” Automated and rapid turnaround follow-up text message responses were developed, containing encouragement and additional information tailored to acknowledge the reply.

**Table 3:** Examples of interactive text messages.

Interactive text sent and patient response		Condition	Automated texts acknowledging response
**Self-efficacy** **—“*Annie-BP*: how confident are you that you can make small daily changes to increase your activity? Text EX 1 (not at all), EX 2 (somewhat) or EX 3 (very) to reply”**			
	EX 1, EX 2	When reply received	“*Annie-BP*: thanks for letting us know. To learn more about exercise opportunities, check out (URL provided)”
	EX 3	When reply received	“*Annie-BP*: That’s great! If you’d still like to learn more about exercise opportunities, check out (URL provided)”
	No reply or reminder	N/A^a^	“*Annie-BP*: how confident are you that you can make small daily changes to increase your activity? Text EX1 (not at all), EX2 (somewhat), or EX3 (very) to reply.”
**Behavioral—“*Annie-BP*: How many DAYS this week have you done a specific exercise other than what you do around the house or at work? Text ACT 0, ACT 1 up to ACT 7 to reply.”**			
	ACT 0, ACT 1, ACT 2	When reply received	“*Annie-BP*: Getting exercise is hard & support helps. Ask your care team: is VA MOVE! right for me? Options include teleMOVE and mobile apps. (URL provided)”
	ACT 3, ACT 4, ACT 5, ACT 6, ACT 7	When reply received	“*Annie-BP*: Good work! To support exercise routines, ask your care team: is VA MOVE! right for me? Options include teleMOVE & mobile apps (URL provided)”
	No reply or reminder	N/A	“*Annie-BP*: How many DAYS this week have you done a specific exercise other than what you do around the house or at work? Text ACT0, ACT1, ACT2 up to ACT7 to reply”

^a^N/A: not applicable.

### Content Influenced by Texting Protocol Structure

Our text message content development was further guided by the development of a structure for a 6-month texting protocol (Multimedia Appendix 1). Each 2-week block addressed a single key concept (Table 1) in hypertension self-management and contained 8 text messages spaced across 14 days. As detailed below, informed by Veteran input, we incorporated a structured combination of the 3 message types (narrative, educational, and interactive) into each block. Texts were designed to be delivered consecutively. The automated response for the first interactive text (delivered at the start of a 2-week block) suggested a website with further information on the content (providing resources for self-guided education); the response for the second interactive text (sent at the end of the 2-week period) provided suggestions for accessing additional VA resources (Table 3). Thus, interactive texts acted as *bookends*; messaging in some instances also built across multiple texts within the 2-week period.

## Results

### Overview

Within the VA texting system *Annie*, we programmed five 6-month text-messaging protocols that included cycles of 3 text message types: (1) narrative messages, (2) nonnarrative educational messages, and (3) 2-way interactive messages assessing self-efficacy and behavior related to hypertension self-management (Multimedia Appendix 2). Each of the protocols corresponds to a single Veteran storyteller, allowing the Veterans to choose the story that most resonates with their own life experiences. Veteran consultant input played a pivotal role in the content and design of our final protocols.

### Incorporating Veteran Consultant Feedback on Cultural Sensitivity of Text Messages

Both Veteran consultants felt strongly that our approach to narrative text development should include direct quotations from our Veteran storytellers as was our original intent. Working directly from professionally typed transcriptions of our Veterans’ stories, we initially crafted texts that sought to adhere closely to the language of the transcription, in the belief that this would be the best way to share the voice of our storytellers. Hence, consistent with the sociolinguistic concept of phonological variants that stay true to actual voices [25], colloquial or informal spellings such as *hafta* and *gotta* were initially included, as were verbs with the final *g* omitted and replaced with an apostrophe (eg, runnin’, eatin’). In an effort to convey the linguistic choices of our Veteran storytellers through our text messages, we also crafted text messages that included sentences with the vernacular features used by the speakers in the delivery of their stories.

RC provided important feedback on these choices. He addressed the subjectivity of our professionally typed transcriptions, pointing out that when he listened, he did not hear *hafta* (“I heard *have to*”) and that transcribing language in this way can reflect the bias of the transcriber. “You should write it how I hear it,” he advised. He emphasized that if we included quotations with colloquial spelling, we might achieve an effect that was the opposite of what we intended. “They’ll think you made a mistake, or they’ll be offended.” RC further pointed out that Veterans receiving our text messages may have personally experienced stigmatization related to their speech patterns, which could influence their interpretation of a text message using nonstandard spelling. Finally, he reminded us of our obligation in seeking to serve the African American Veteran community: to keep in mind that some of our intended text message recipients have likely suffered from disparities in access to high-quality education, and that we should use standard spelling to avoid perpetuating these disparities through our texts. Taking all these points into account, we modified all our narrative text messages to adopt standard spelling while trying to keep the cadence of the Veteran voice.

### Incorporating Veteran Consultant Feedback on Text-messaging Protocol Structure

Our Veteran consultant (RC) helped define the strategy for integrating educational and narrative texts into the protocol. He suggested that, for each key concept in hypertension self-management, we begin by providing educational text messages for 1 week without referencing the Veteran storyteller (*to see if they can do it themselves* and *give them a little space*). He suggested bringing the storyteller’s voice back into the conversation after the first week. Thus, following a week of educational texts, our protocol includes a week of narrative texts intended to draw recipients back into the story, using messages that have personal relevance to *continue the conversation*. RC also provided specific feedback on the order in which narrative messages should be presented, seeking to maintain a narrative arc within the week wherever possible.

By incorporating Veteran feedback on text message content and protocol structure, our team refined our overarching structure for a 6-month texting protocol. We outlined a 26-week protocol with 9 key content area blocks. We repeated 2 content areas (salt intake and taking medicine) using identical content when repeating blocks. These were chosen for repetition because of their wide applicability across our intended target population and abundant content from Veteran story transcripts. To further support opportunities for self-reflection and to periodically renew engagement, we included a check-in week after every 4 key concept blocks (every 8 weeks); this check-in week was composed entirely of interactive texts. We intended this check-in primarily for motivational purposes but secondarily planned to use it to assess the sustenance of self-management behaviors. Each check-in week began with the same overarching assessment of self-efficacy (“How confident are you in managing your BP? Text CONF1 (not at all confident), CONF2 (somewhat), or CONF3 (very) to reply”) and then repeated 3 behavioral assessments used in the preceding 8 weeks, inviting reflection on performance of behaviors over *this week* (eg, asking participants to reflect on their performance over the check-in week).

With input from our Veteran consultant, we determined that the timing of the texts would be 11 AM and 3 PM to account for off-shift as well as day shift workers. Pilot testing is planned to ascertain the acceptability of using the default timing (as opposed to customized) and to assess the usability of the interactive components of the texting protocol.

## Discussion

### Principal Findings

Our goal was to design a text-messaging protocol that preserved the voice of our storytellers while conveying authentic and respectful messages to African American Veterans in support of their hypertension self-management. We used 3 complementary strategies to accomplish this goal. First, we built directly on our previous work on the use of storytelling to support behavior change. The use of the VA Annie texting platform for research purposes is still a nascent field of study. Our second strategy was therefore focused on understanding and responding to the constraints imposed by the Annie system while taking advantage of the opportunities afforded by this nationally available system. This adaptation was made possible through the guidance offered by a few VA researchers experienced in the use of this platform as well as from VA operational partners. Finally, our third strategy was to pay careful attention to the inputs provided by our Veteran consultants to design both content and a texting structure informed by cultural considerations.

This work builds on previous work on the use of storytelling to support hypertension management in African Americans. Previous research has advanced the theory and empirical evidence for using peer narrative communication (storytelling) to promote patient engagement and hypertension self-management [26]. Schoenthaler et al [26] combined the information-motivation-behavioral theory with qualitative feedback from African Americans with hypertension to tailor their mobile health intervention. We were informed by SCT in our development of both text message content and texting protocols. Interactive text messages were designed to *bookend* a 2-week focus on a key content area. The 2-weeks begin with an interactive text assessing self-efficacy, following which we provided one week of educational messages intended to support behavioral capability and self-regulation using friendly and encouraging language. In the second week, narrative messages convey the voice and sentiments of the storytellers with the intent of incentivizing and reinforcing health behaviors by referencing the actions of participants’ preferred storytellers. SCT focuses on interactions among people, their behavior, and their environment. We used this approach to connect the hypertension control strategies of the storytellers to those of the participants in the context of their daily routines.

In a randomized trial conducted in a safety-net hospital in Birmingham, Alabama, an intervention based on video-recorded stories by African Americans led to significant improvements in blood pressure at 3 months among those with uncontrolled hypertension [27]. In our previous work, we translated these findings to the VA, developing and testing video-recorded Veteran narratives shown to African American Veterans during VA clinic visits [12]. We demonstrated significant differences in intention to change hypertension self-management immediately after viewing the stories; the effects on blood pressure were not sustained, with 6-month outcomes revealing only modest benefit (3.1 mm Hg) versus control (*P*=.06). Our findings highlight the need to focus on longitudinal support to sustain the storytelling effect, which prompted us to explore the opportunities presented by the nationally available VA texting platform, Annie.

Similarly, others have developed text-messaging protocols tailored to a target population [15,26,28-30]. Including the target population in the design can help ensure that the intervention is culturally relevant [26]. Tailoring messages that align with theory and are tailored to be culturally relevant show promise to be more effective than standard care [28]. Barsky et al [29] developed a text-messaging intervention comparing active hypertension management versus passive health behavior messages, and found that this approach helped patients in remote areas feel connected and supported [29]. Others have found text messages supporting hypertension control to be both a feasible and an acceptable format for African Americans [15], with the potential to improve adherence [31]. Other studies working to design text-messaging protocols to support African Americans with hypertension sought to design culturally appropriate messaging, a goal that we shared. Our strategy aligned with those of other studies insofar as we sought inputs from our end-user population (Veteran consultant input); other studies made modifications to enhance the cultural sensitivity of the messages [29] and elicited end-user feedback through focus groups [15] or qualitative interviews [26]. According to our review of the literature, our approach to designing messages to support hypertension self-management in African Americans is unique in its incorporation of Veteran’s voice and story. In addition, longitudinal data, such as those proposed in our study, are still required [26].

Constraints placed by the Annie texting platform required creativity to improve our texting design in some instances; in other cases, these platform limitations are expected to introduce challenges at the implementation stage. The requirement that the word *Annie* be included in every text for identification purposes prompted us to *brand* our emails (each begins with *Annie-BP*) which may facilitate recognition and decrease the likelihood of these messages being confused with any others. With a limited character count, this requirement occasionally forced us to cut down messaging or spread a concept over several messages (spaced across days). Interactive texts have strict requirements for formatting responses (and lack the ability to be customized to provide automated guidance when the response is incorrect). This requirement is expected to pose challenges for less technically proficient Veterans, particularly when texting protocols must be initiated virtually. There is currently no easy way to change the time of delivery of all messages across a multi message, multiday protocol; timing must be changed manually (message-by-message) to accommodate Veteran preferences, introducing the possibility of programming errors when implemented in real time (eg, during enrollment of a Veteran). We are working with our Veteran consultants to select times with broad acceptability and have provided this feedback to VA operational partners as a possible update to Annie that would allow for more Veteran-centered choices in future work.

Inputs from our consultants, both African American Veterans, were instrumental in the development of this text message content and protocol. The development of culturally sensitive messaging is critical for health care and public health messages [32]. Mobile health programs for behavior change reflect opportunities to reach underserved populations in real time in a relevant, sustainable, and scalable manner [33]. Amid the current COVID-19 pandemic, text message programs that seek to convey personally relevant and culturally sensitive messages of support represent a potentially valuable way to reach out to otherwise isolated Veterans. If such messages help our Veterans feel connected to their health care team, this delivery may be an important way to enhance trust during this difficult time and could have additional long-term benefits.

Our protocol is being piloted using methods adapted in the setting of the current COVID-19 pandemic; we are reaching out to the Veterans and delivering the intervention to them virtually rather than following our original study design (ie, we are no longer asking them to come in-person to the clinic). Participants will be invited to watch 5 storyteller videos and then select their preferred storyteller. This selection will guide the assignment of the texting protocol. After the pilot and refinement of the protocol as needed, we will conduct a 2-site randomized controlled trial to determine if our *continuing the conversation* protocol can improve not only intentions, but also blood pressure control. If successful, our storyteller video and texting package can provide access to culturally sensitive health care messaging for African American Veterans who have difficulty controlling their blood pressure but who are unable to come in-person to the clinic.

### Conclusions

Incorporating quotations from the stories of African American Veterans and including African American Veteran consultants on our team allowed us to create culturally sensitive, 6-month text-messaging protocols aimed at supporting effective hypertension self-management through greater engagement and behavioral change.

### Practice Implications

Text-messaging platforms provide important tools to continue conversations and provide sustained cues for behavior change. By addressing inequities in health care delivery and access and by supporting the promotion of healthy behaviors, our planned intervention seeks to address several key VA priorities. We hope that our findings will also have broader applicability and reach beyond our Veteran population. A memorable story stays with the listener as an inspiration and guide. Our *continuing the conversation* video and texting package aims to weave the storyteller’s messages into the everyday life of the text recipients, reminding and reinforcing them as our recipients engage in the numerous daily decisions that will impact their blood pressure and their lives.

## Appendix

Multimedia Appendix 1 Example 6-month texting protocol.

Multimedia Appendix 2 Continuing the Conversation 9-week example protocol.

## Abbreviations

SCT: Social Cognitive Theory
VA: Veterans Affairs
