# Supplementary figures and images for: Incorporating African American Veterans’ Success Stories for Hypertension Management: Developing a Behavioral Support Texting Protocol
Source: JMIR Res Protoc. 2021 Dec 1;10(12):e29423. doi: 10.2196/29423 (PMC8686408; doi:10.2196/29423)

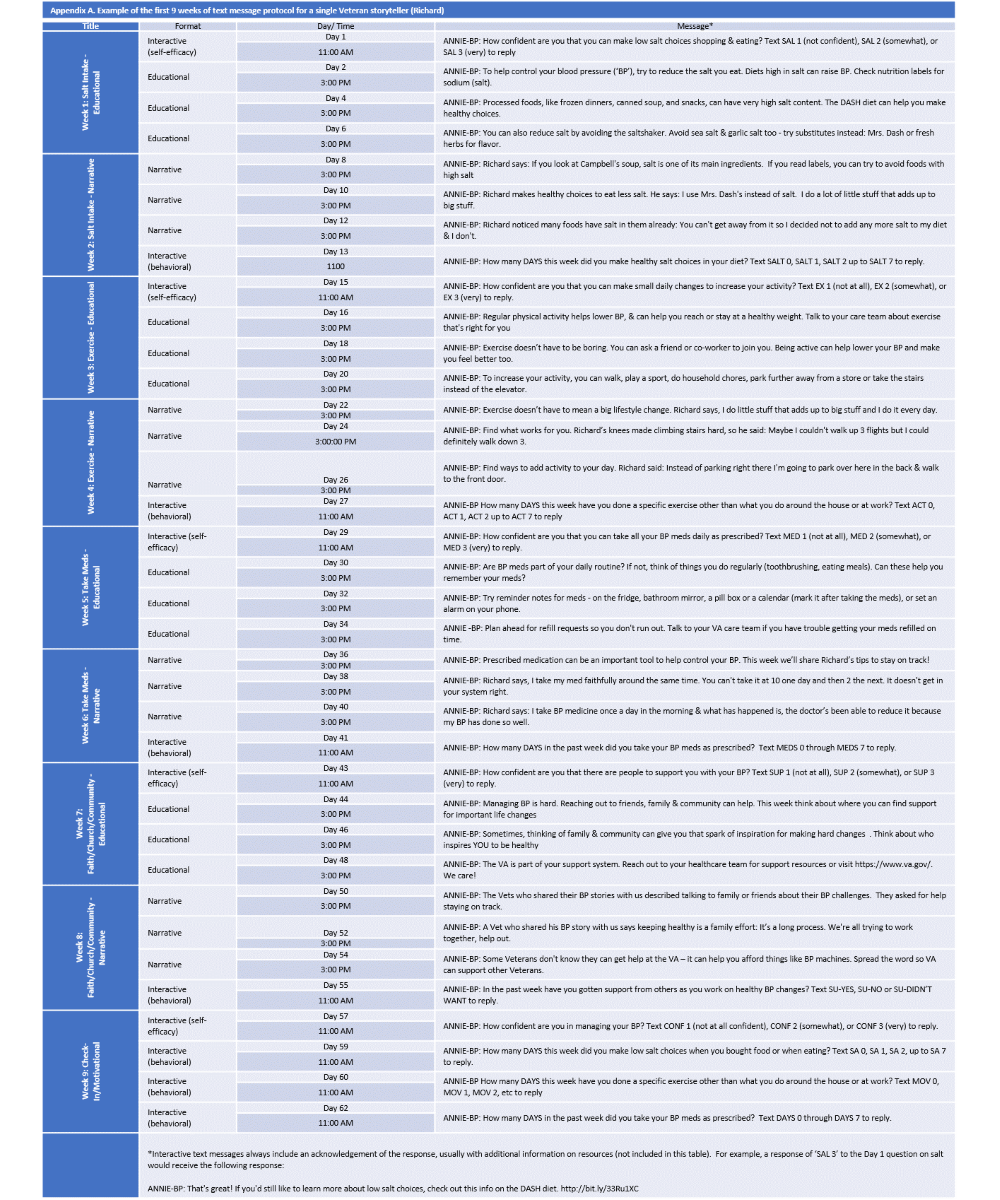

Supplement: Multimedia Appendix 1 [file resprot_v10i12e29423_app1.png]
