# Supplementary material for: Incorporating African American Veterans’ Success Stories for Hypertension Management: Developing a Behavioral Support Texting Protocol
Source: JMIR Res Protoc. 2021 Dec 1;10(12):e29423. doi: 10.2196/29423 (PMC8686408; doi:10.2196/29423)
Supplement: Multimedia Appendix 2 [file resprot_v10i12e29423_app2.docx]

| **Appendix A. Example of the first 9 weeks of text message protocol for a single Veteran storyteller (Richard)** | | | |
| --- | --- | --- | --- |
|  |  |  |  |
| **Title** | **Format** | **Day/ Time** | **Message*** |
| **Week 1: Salt Intake - Educational** | Interactive  (self-efficacy) | Day 1 | ANNIE-BP: How confident are you that you can make low salt choices shopping & eating? Text SAL 1 (not confident), SAL 2 (somewhat), or SAL 3 (very) to reply |
|  |  | 11:00 AM |  |
|  | Educational | Day 2 | ANNIE-BP: To help control your blood pressure (‘BP’), try to reduce the salt you eat. Diets high in salt can raise BP. Check nutrition labels for sodium (salt). |
|  |  | 3:00 PM |  |
|  | Educational | Day 4 | ANNIE-BP: Processed foods, like frozen dinners, canned soup, and snacks, can have very high salt content. The DASH diet can help you make healthy choices. |
|  |  | 3:00 PM |  |
|  | Educational | Day 6 | ANNIE-BP: You can also reduce salt by avoiding the saltshaker. Avoid sea salt & garlic salt too - try substitutes instead: Mrs. Dash or fresh herbs for flavor. |
|  |  | 3:00 PM |  |
| **Week 2: Salt Intake - Narrative** | Narrative | Day 8 | ANNIE-BP: Richard says: If you look at Campbell's soup, salt is one of its main ingredients. If you read labels, you can try to avoid foods with high salt |
|  |  | 3:00 PM |  |
|  | Narrative | Day 10 | ANNIE-BP: Richard makes healthy choices to eat less salt. He says: I use Mrs. Dash's instead of salt. I do a lot of little stuff that adds up to big stuff. |
|  |  | 3:00 PM |  |
|  | Narrative | Day 12 | ANNIE-BP: Richard noticed many foods have salt in them already: You can't get away from it so I decided not to add any more salt to my diet & I don't. |
|  |  | 3:00 PM |  |
|  | Interactive (behavioral) | Day 13 | ANNIE-BP: How many DAYS this week did you make healthy salt choices in your diet? Text SALT 0, SALT 1, SALT 2 up to SALT 7 to reply. |
|  |  | 1100 |  |
| **Week 3: Exercise - Educational** | Interactive  (self-efficacy) | Day 15 | ANNIE-BP: How confident are you that you can make small daily changes to increase your activity? Text EX 1 (not at all), EX 2 (somewhat), or EX 3 (very) to reply. |
|  |  | 11:00 AM |  |
|  | Educational | Day 16 | ANNIE-BP: Regular physical activity helps lower BP, & can help you reach or stay at a healthy weight. Talk to your care team about exercise that's right for you |
|  |  | 3:00 PM |  |
|  | Educational | Day 18 | ANNIE-BP: Exercise doesn’t have to be boring. You can ask a friend or co-worker to join you. Being active can help lower your BP and make you feel better too. |
|  |  | 3:00 PM |  |
|  | Educational | Day 20 | ANNIE-BP: To increase your activity, you can walk, play a sport, do household chores, park further away from a store or take the stairs instead of the elevator. |
|  |  | 3:00 PM |  |
| **Week 4: Exercise - Narrative** | Narrative | Day 22 | ANNIE-BP: Exercise doesn’t have to mean a big lifestyle change. Richard says, I do little stuff that adds up to big stuff and I do it every day. |
|  |  | 3:00 PM |  |
|  | Narrative | Day 24 | ANNIE-BP: Find what works for you. Richard’s knees made climbing stairs hard, so he said: Maybe I couldn't walk up 3 flights but I could definitely walk down 3. |
|  |  | 3:00:00 PM |  |
|  | Narrative | Day 26 | ANNIE-BP: Find ways to add activity to your day. Richard said: Instead of parking right there I'm going to park over here in the back & walk to the front door. |
|  |  | 3:00 PM |  |
|  | Interactive (behavioral) | Day 27 | ANNIE-BP How many DAYS this week have you done a specific exercise other than what you do around the house or at work? Text ACT 0, ACT 1, ACT 2 up to ACT 7 to reply |
|  |  | 11:00 AM |  |
| **Week 5: Take Meds - Educational** | Interactive (self-efficacy) | Day 29 | ANNIE-BP: How confident are you that you can take all your BP meds daily as prescribed? Text MED 1 (not at all), MED 2 (somewhat), or MED 3 (very) to reply. |
|  |  | 11:00 AM |  |
|  | Educational | Day 30 | ANNIE-BP: Are BP meds part of your daily routine? If not, think of things you do regularly (toothbrushing, eating meals). Can these help you remember your meds? |
|  |  | 3:00 PM |  |
|  | Educational | Day 32 | ANNIE-BP: Try reminder notes for meds - on the fridge, bathroom mirror, a pill box or a calendar (mark it after taking the meds), or set an alarm on your phone. |
|  |  | 3:00 PM |  |
|  | Educational | Day 34 | ANNIE -BP: Plan ahead for refill requests so you don't run out. Talk to your VA care team if you have trouble getting your meds refilled on time. |
|  |  | 3:00 PM |  |
| **Week 6: Take Meds - Narrative** | Narrative | Day 36 | ANNIE-BP: Prescribed medication can be an important tool to help control your BP. This week we’ll share Richard’s tips to stay on track! |
|  |  | 3:00 PM |  |
|  | Narrative | Day 38 | ANNIE-BP: Richard says, I take my med faithfully around the same time. You can't take it at 10 one day and then 2 the next. It doesn't get in your system right. |
|  |  | 3:00 PM |  |
|  | Narrative | Day 40 | ANNIE-BP: Richard says: I take BP medicine once a day in the morning & what has happened is, the doctor’s been able to reduce it because my BP has done so well. |
|  |  | 3:00 PM |  |
|  | Interactive (behavioral) | Day 41 | ANNIE-BP: How many DAYS in the past week did you take your BP meds as prescribed?  Text MEDS 0 through MEDS 7 to reply. |
|  |  | 11:00 AM |  |
| **Week 7: Faith/Church/Community - Educational** | Interactive (self-efficacy) | Day 43 | ANNIE-BP: How confident are you that there are people to support you with your BP? Text SUP 1 (not at all), SUP 2 (somewhat), or SUP 3 (very) to reply. |
|  |  | 11:00 AM |  |
|  | Educational | Day 44 | ANNIE-BP: Managing BP is hard. Reaching out to friends, family & community can help. This week think about where you can find support for important life changes |
|  |  | 3:00 PM |  |
|  | Educational | Day 46 | ANNIE-BP: Sometimes, thinking of family & community can give you that spark of inspiration for making hard changes  . Think about who inspires YOU to be healthy |
|  |  | 3:00 PM |  |
|  | Educational | Day 48 | ANNIE-BP: The VA is part of your support system. Reach out to your healthcare team for support resources or visit https://www.va.gov/. We care! |
|  |  | 3:00 PM |  |
| **Week 8: Faith/Church/Community - Narrative** | Narrative | Day 50 | ANNIE-BP: The Vets who shared their BP stories with us described talking to family or friends about their BP challenges. They asked for help staying on track. |
|  |  | 3:00 PM |  |
|  | Narrative | Day 52 | ANNIE-BP: A Vet who shared his BP story with us says keeping healthy is a family effort: It’s a long process. We're all trying to work together, help out. |
|  |  | 3:00 PM |  |
|  | Narrative | Day 54 | ANNIE-BP: Some Veterans don't know they can get help at the VA – it can help you afford things like BP machines. Spread the word so VA can support other Veterans. |
|  |  | 3:00 PM |  |
|  | Interactive (behavioral) | Day 55 | ANNIE-BP: In the past week have you gotten support from others as you work on healthy BP changes? Text SU-YES, SU-NO or SU-DIDN’T WANT to reply. |
|  |  | 11:00 AM |  |
| **Week 9: Check-In/Motivational** | Interactive (self-efficacy) | Day 57 | ANNIE-BP: How confident are you in managing your BP? Text CONF 1 (not at all confident), CONF 2 (somewhat), or CONF 3 (very) to reply. |
|  |  | 11:00 AM |  |
|  | Interactive (behavioral) | Day 59 | ANNIE-BP: How many DAYS this week did you make low salt choices when you bought food or when eating? Text SA 0, SA 1, SA 2, up to SA 7 to reply. |
|  |  | 11:00 AM |  |
|  | Interactive (behavioral) | Day 60 | ANNIE-BP How many DAYS this week have you done a specific exercise other than what you do around the house or at work? Text MOV 0, MOV 1, MOV 2, etc to reply |
|  |  | 11:00 AM |  |
|  | Interactive (behavioral) | Day 62 | ANNIE-BP: How many DAYS in the past week did you take your BP meds as prescribed?  Text DAYS 0 through DAYS 7 to reply. |
|  |  | 11:00 AM |  |
|  | *Interactive text messages always include an acknowledgement of the response, usually with additional information on resources (not included in this table). For example, a response of ‘SAL 3’ to the Day 1 question on salt would receive the following response:  ANNIE-BP: That's great! If you'd still like to learn more about low salt choices, check out this info on the DASH diet. http://bit.ly/33Ru1XC | | |
